# Analysis of Surgical Stabilization Results of Radial Head Fractures

**DOI:** 10.3390/jcm14041336

**Published:** 2025-02-17

**Authors:** Paweł Niewczas, Piotr Piekarczyk, Łukasz Jacuniak, Dawid Lewandowski, Tomasz Ząbkowski, Kamil Ciechan, Piotr Cieślik

**Affiliations:** 1 Department of Traumatology and Orthopedics, Military Institute of Medicine—National Research Institute, 128 Szaserów Street, 04-141 Warsaw, Poland; pniewczas@wim.mil.pl (P.N.); piotr@msnet.pl (P.P.); ljacuniak@wim.mil.pl (Ł.J.); dlewandowski@wim.mil.pl (D.L.); pcieslik@wim.mil.pl (P.C.); 2 Department of Urology, Military Institute of Medicine–National Research Institute, 128 Szaserów Street, 04-141 Warsaw, Poland; tzabkowski@wim.mil.pl; 3 Trainee Attorney-at-Law, Warsaw Bar Association, 01-014 Warsaw, Poland

**Keywords:** radial head fractures, Mason classification, open reduction and internal fixation

## Abstract

**Background/Objectives**: According to the modified Mason classification, radial head fractures can be treated with open reduction and internal fixation (ORIF), radial head arthroplasty, or resection. This study by the Department of Traumatology and Orthopedics of the Military Institute of Medicine aimed to compare the clinical and radiological outcomes of fractures treated with ORIF. **Methods**: This retrospective study analyzed 55 patients with radial head fractures treated between April 2020 and February 2023. Fractures were classified using Mason system as 15 type II, 26 type III, and 14 type IV. Clinical outcomes were evaluated using the VAS for pain, Broberg–Morrey scale, and the DASH questionnaire, alongside assessments of range of motion, grip strength, and elbow stability. Follow-up radiographs examined bone union, bone fragments displacement, degenerative changes and periarticular ossification. **Results**: The mean follow-up period was 21.0 ± 10.2 months. There were no statistically significant differences in VAS scale results for Mason types II, III, and IV fractures (4.0 vs. 6.0 vs. 5.0, respectively; *p* = 0.825), the Broberg–Morrey scale (82.0 ± 15.2 vs. 80.9 ± 15.5 vs. 84.2 ± 15.1, respectively; *p* = 0.845), or the DASH questionnaire (10.0 vs. 11.7 vs. 17.5, respectively; *p* = 0.937). Mean extension deficit and supination angles were at the limit of statistical significance (*p* = 0.076 and *p* = 0.051). No cases of lateral instability were observed, whereas medial instability was seen in only one case. Bone union was observed in 97.5% of cases, with elbow joint osteoarthritis and periarticular ossification in 15.0% and 45.0% of cases, respectively. **Conclusions**: Mason type II, III and IV radial head fractures treated with open reduction and internal fixation showed good functional and radiological outcomes with rare complications, including degenerative changes, periarticular ossifications and nonunion.

## 1. Introduction

Radial head fractures account for 1.5–4% of all fractures in adults [1,2]. The division of these fractures is based on the Mason classification [3], with a modification by Johnston [4]. The criteria for the division are the degree of fragmentation and displacement of fragments. According to Mason classification, type I fractures (non-displaced or with a displacement up to 2 mm) are most often treated non-operatively, with good treatment outcomes [5,6]. Simple fractures displaced >2 mm are classified as type II fractures. Due to the high risk of mechanical block with limited range of motion of the elbow joint, surgical treatment is most performed. According to some reports, treatment results are comparable to those of non-operative treatment [7,8].

Type III includes comminuted fractures involving the entire surface of the radial head, whereas type IV includes fractures coexisting with the elbow joint dislocation. In the past, the only treatment for such fractures was radial head resection, which is no longer commonly practiced [9]. Types III and IV fractures are treated with open reduction and internal fixation (ORIF) or radial head arthroplasty (RHA) when ORIF is contraindicated [10]. The most commonly chosen method for treating type III fractures is RHA [11] though no consensus has been reached yet. The choice between ORIF or RHA often depends on surgeon’s experience and preferences.

In the Department of Traumatology and Orthopedics of the Military Institute of Medicine, ORIF is preferred for the treatment of types II, III, and IV fractures, while other methods are not practiced due to surgeons’ individual experience and previous good clinical results [12]. Most of available publications on radial head fractures compare the results of treatment between different surgical methods for one type of fracture according to Mason classification [7,11,13,14,15,16,17,18,19,20]. However, there is a limited number of studies comparing the outcomes of different Mason type fractures treated with the same method (ORIF). We believe that the results of treatment are not determined only by the choice of surgical method but also by accompanying injuries and their management. Helmstetter et al. [21] suggested that minimally invasive approach and soft tissue repair reduced unsatisfactory results of ORIF, even in cases with comminution of radial head.

This study aimed to compare the clinical, functional, and radiological results of surgical treatment of type II, III, and IV radial head factures according to the Mason classification using open reduction internal fixation.

## 2. Materials and Methods

This retrospective study included patients with radial head fractures hospitalized in the Department of Traumatology and Orthopedics of the Military Institute of Medicine between April 2020 and February 2023.

Patients with polytrauma, terrible triad of the elbow and those treated nonoperatively were excluded. Fifty-three patients who underwent open reduction internal fixation were included in this study.

Data obtained from the Asseco Medical Management Solutions (AMMS) in-hospital information system (age at the time of injury, sex, side of the limb involved, dominant limb, mechanism of injury, associated fractures in the upper limb, occupational status, sporting activity, and smoking) were included in the analysis. Information regarding intraoperative observations was obtained from surgical descriptions (number of intermediate fragments, accompanying articular cartilage exfoliation of the radial head, assessment of ligamentous damage to the elbow joint i.e., LCL and MCL, assessment of bony defects of the radial head, and postoperative neurovascular disorders). Data from the postoperative follow-up visits, duration of immobilization after surgery, and postoperative complications were analyzed.

The affected limb was placed on a lateral table during surgery. We used Esmarch’s tourniquet in most cases. Either a posterolateral approach (known as Kocher approach) was usually chosen. The radial head and other intraarticular structures were assessed. Then a stability of elbow in full extension and 30° flexion was examined under fluoroscopy. The next step was to reduce the fracture, most often in order from the largest to the smallest bone fragments. If a radial head was anatomically reduced, then it was temporary fixed with K wires inserted parallel to the cartilage. If the reduction was stable, then the ASNIS Micro cannulated screws (Asnis Micro Cannulated Screw System; Stryker Inc., Kalamazoo, MI, USA) were tighten along the K wires. If the reduction stability was not clear, a fracture was also fixated with a buttress plate (VariAx Hand Plating System; Stryker Inc., Kalamazoo, MI, USA). During tightening screws into bone, care must be taken not to lose the reduction. In case of capitulum fracture, another one cannulated screw (Asnis Micro Cannulated Screw System; Stryker Inc., Kalamazoo, MI, USA) was used to fixate the fracture. Delamination of articular cartilage was usually managed with tissue adhesive. Ligament ruptures were repaired with suture anchors (JuggerKnot Soft Anchor System; Zimmer Biomet, Warsaw, IN, USA). The anchors were placed in lateral or medial epicondyle due to LCL complex or MCL rupture, respectively. Ligament fragments were fixed with Krackow suture.

Postoperatively, patients were immobilized with a cast in 90° flexion and neutral forearm rotation for about 2 weeks. After that, passive ROM exercises (including gravity-assisted and contralateral-arm-assisted) were implemented in physiotherapy protocol for about 2 weeks. Patients were then gradually encouraged to perform active elbow exercises under physiotherapist supervision until 6 weeks after surgery. There was neither indomethacin nor any other nonsteroidal anti-inflammatory drug was used for periprosthetic ossification prevention.

Long-term treatment outcomes were assessed based on the data obtained during follow-up visits. Visits were conducted at an outpatient orthopedic clinic. The patients completed visual analog scale (VAS) pain questionnaire and Disabilities of Arm, Shoulder, and Hand (DASH) functional assessment questionnaire. Physical examination was performed by senior assistant from the Department of Traumatology and Orthopedics. Flexion and extension angles of the elbow joint, as well as the pronation and supination of the forearm, were measured using a goniometer. Elbow joint stability was assessed in full extension and 30° flexion. Dynamometric measurement of grip strength of both operated and uninjured limbs was analyzed, and the operated-to-uninjured grip strength ratio was used in the analysis. The Broberg–Morrey scale was used for clinical assessment (scores include very good 95–100 points, good, 80–94 points, sufficient 60–79 points, and bad <60 points).

**Figure 1:**
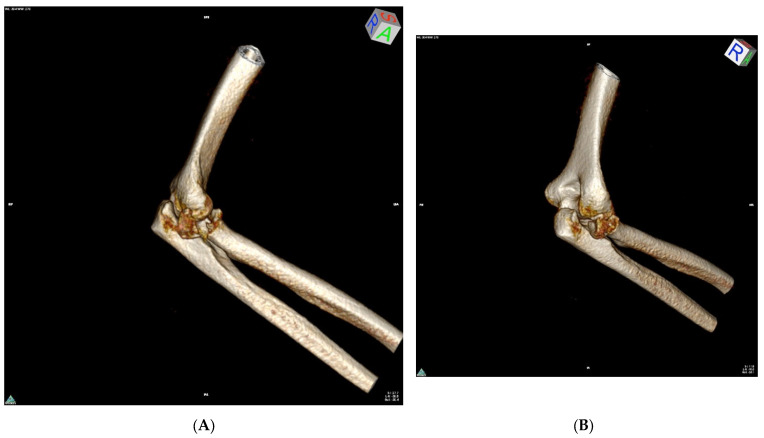
Example of a CT scan (three-dimensional volume reconstruction) showing a Mason type III radial head fracture with significant fracture displacement—P.O. 35y (**A**,**B**).

**Figure 2:**
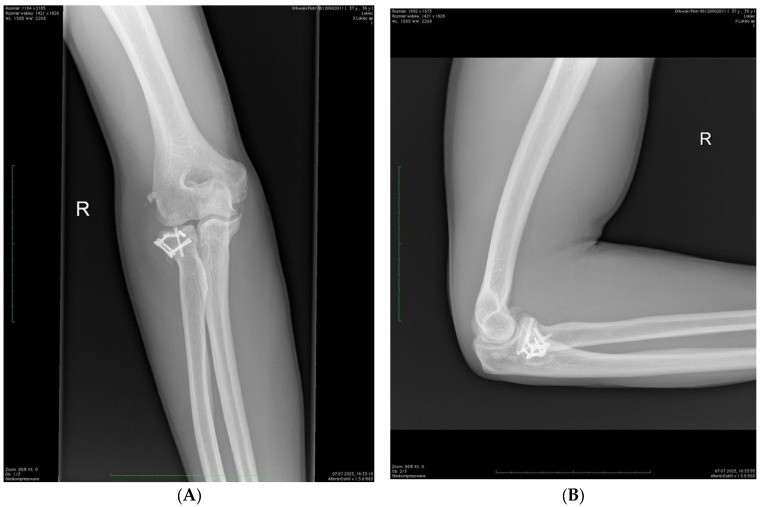
Control X-ray in the same patient 23 months after surgery—AP (**A**) and lateral (**B**) projections. Complete bone union and asymptomatic periarticular ossification (Hastings–Graham 1).

Radiological examinations from the Digital Imaging and Communications in Medicine database were analyzed i.e., X-rays and CT scans after injury (Figure 1), X-rays after surgery and X-rays at the last follow-up visit (Figure 2). Bone union, fracture displacement, periarticular ossification (the Hastings–Graham scale), elbow osteoarthritis, and signs of avascular necrosis of radial head were assessed.

The analysis was performed using R software (version 4.1.2). Basic descriptive statistics were used to describe numerical variables. Categorical variables are presented as number of observations and percentage of group participation. Normality of the distributions was checked with the Shapiro–Wilk test and verified using the skewness and kurtosis coefficients. Comparisons between groups were made using analysis of variance analysis and the Kruskal–Walli’s test for numerical variables and Pearson’s chi-square and Fisher’s exact tests for categorical variables. Throughout the analysis, *p* values less than 0.05 were regarded as evidence for statistical significance.

## 3. Results

The analyzed group included 55 cases from 53 patients. Age ranged between 18–78 years, with a mean of 43.5 ± 15.1 years. In two patients, the fracture occurred bilaterally, with one patient having a simultaneous fracture and the other having fractures of the individual limbs occurring at different times. More than half of the participants were women (56.6%). Average body mass index was 28.4 ± 5.9 kg/m^2^. Smoking was observed in five cases. Office work is the most common occupation. In more than half of the patients, participation in sports was declared (57.1%).

The most common injury occurred as a result of a fall on an extended limb (61.8%). In one patient, the injury resulted from a fall on the elbow-bent limb. In 47.1% of the patients, the fracture involved the dominant limb. No vascular injuries were observed in the study group, whereas one patient showed signs of post-traumatic brachial plexus injury. The general characteristics and occupational status of the patients are shown in Table 1.

According to the Mason scale, 27.3% of the cases were classified as type II and 47.3% as type III. A fracture with concomitant dislocation of the elbow joint (type IV according to the modified Mason scale) was diagnosed in 25.5% cases. An open fracture was diagnosed in one patient. Comminuted fractures (with > 2 fragments, including the shaft of the radius as a single fragment) were diagnosed in 61.8% cases. Articular cartilage degeneration occurred in nine cases. Lateral collateral ligament complex injury (LCL) was observed in 76.4 per cent cases, while the medial collateral ligament rupture (MCL) affected 20.0% cases. A concurrent capitellum fracture occurred in 21.8% cases. In 76.4% cases, the fracture was stabilized with ASNiS cannulated screws (Stryker) and anatomic buttress plate VariAx (Stryker), in 20.0% cases with ASNiS 2.0 cannulated screws (Stryker), and only in two cases with anatomic VariAx plate (Stryker).

Postoperatively, immobilization was used for 9–49 days, with a mean of 21.7 ± 10.4 days.

Characteristics of the groups during the operative period according to fracture type are presented in Table 2.

Thirty-nine patients attended the follow-up visits, yielding data from 41 cases. Follow-up time varied between 4.0–40.0 months, with a mean of 21.0 ± 10.2 months. The mean values and ranges of the individual parameters are listed in Table 3.

The mean visual analog scale (VAS) pain score was 13.9 ± 20.6 and no statistically significant differences were observed among cases with individual fracture types (*p* = 0.825). Similarly, no statistically significant differences were observed between the groups for the DASH and Broberg-Morrey functional scale scores (*p* = 0.937 and *p* = 0.845, respectively). The mean scores for these scales in the study group were 20.8 ± 22.4 and 82.0 ± 15.0, respectively. The results of the clinical assessments are presented in Table 4. Very good and good scores were obtained in 83.0% of the cases (41.5% for each score). A sufficient score affected 12.2% of the patients. Only two patients had poor treatment outcomes.

The mean flexion angle at the elbow joint was 134.6 ± 12.1 degrees, while the mean extension deficit was 11.6 ± 15.7 degrees. While the flexion angle values in different fracture types did not differ significantly, the extension deficit angle values varied at the limit of statistical significance (*p* = 0.076). Regarding rotational movements of the forearm, the mean pronation angle was 79.5 ± 16.3° and supination 58.3 ± 28.1 degrees. The supination angles for each fracture type were also at the limit of statistical significance (*p* = 0.051). The operated-to-uninjured grip strength ratio averaged 80.4 ± 24.5% and did not differ significantly between groups. No cases showed features of lateral elbow instability, whereas medial instability was observed in one patient with a Mason type IV fracture.

Radiological results of treatment are presented in Table 3. The results were obtained for 40 patients. Follow-up radiographs were waived for one patient due to pregnancy. Bone union was observed in 97.5% of the patients. No secondary fragments displacement was observed. Osteoarthritis was observed in 15.0% cases. Periarticular ossification occurred in 45.0% cases, six of which were asymptomatic (type 1 according to the Hastings–Graham scale). Only one patient had ankylosis of the elbow joint with significant limitations in flexion and extension movements (type 3a). Considering the division of fracture types according to the modified Mason scale, the individual results did not differ significantly.

## 4. Discussion

Despite improvements in surgical techniques, comminuted radial head fractures remain challenging [22,23,24]. No clear guidelines for the management of such fractures have emerged to date. The criteria used by Ring et al. [25], who recommended the use of ORIF methods in fractures with fewer than three intermediate fractures and the use of RHA in complex fractures, have been widely adopted. In contrast, Burkhart et al. [26] recommend ORIF in all cases where anatomical reduction and stable fixation is achievable.

The present study showed that the clinical results of radial head fractures treatment with ORIF methods were satisfactory. There were 41.5% very good and 41.5% good responses. There were no statistically significant differences in the treatment results for individual fracture types. Several studies reported similar results [21,22,24,27,28,29,30,31,32,33,34,35]. Walsh et al. [36] reported no difference in outcomes between simple and complex radial head fractures treated with ORIF. Additionally, Müller et al. showed that ORIF with a plate or screws provided good results [37]. In contrast, better functional outcomes were obtained in patients with type II fractures than in those with type III fractures.

Radiographic examination revealed bone union in 97.5% of patients. Displacement of the fragments was not observed. Nonunion occurred in only one patient with morbid obesity (body mass index, 52.0 kg/m^2^). The patient experienced little pain, and the supination movement of the forearm was limited. We found no reports in the literature supporting the hypothesis that obesity affects nonunion of the radial head. Post-traumatic osteoarthritis occurred in 15.0% cases and periarticular ossification in 45.0% cases.

Importantly, 33.3% of patients with periarticular ossification had no functional impairment of the operated limb (grade 1 according to Hastings–Graham). These results did not differ from those obtained by other authors [17,21,22,24,38,39]. In some studies, post-traumatic osteoarthritis and periarticular ossifications occurred less frequently [15,23,27,29,31,33,40]. In the present study, we did not use indomethacin or radiotherapy to prevent periarticular ossification.

Most of available studies present a comparative analysis of the results of radial head fractures treatment using different surgical methods. The results of these analyses vary. Some studies have shown the superiority of ORIF over other treatment modalities [14,15,19,27,41]; however, in the majority of publications, RHA yields better clinical results [11,13,16,42,43] or the results do not differ significantly [9,17,18,40]. To the best of our knowledge, only one meta-analysis has demonstrated the superiority of ORIF over other treatments [44]. In the other meta-analyses, radial head prosthesis yields better functional outcomes and fewer complications compared to ORIF [16,20]. However, RHA has some limitations (risk of periprosthetic osteolysis, implant loosening, and displacement). Additionally, the implantation of an overly large endoprosthesis may result in overstuffing [45]. Furthermore, the radial bone head can show substantial morphologic variations, making it difficult to fit an implant that is perfectly congruent with the articular surface of the elbow joint [46].

When comparing the treatment results of different surgical methods, the continuous technological progress and development of biomedical engineering should be considered [33,47]. Gruszka et al. [38] emphasize that the use of modern anatomical locking plates provides good results in the treatment of fractures of the head and neck of the radial bone, even in comminuted fractures associated with damage to other structures of the elbow joint. The authors used both rim and buttress plates but showed no statistically significant differences in treatment outcomes. Good results in radial head fractures treatment with cannulated screw stabilization using the ‘tripod’ technique have also been described [39].

The associated injuries and stability of elbow joint in radial head fractures are important issues. Many authors emphasize the role of radial head as a secondary stabilizer of the elbow joint against valgus forces and axial loading [10,43,48,49]. Rhyou et al. [50] believed that radial head fractures are always accompanied by collateral ligaments rupture and comminuted fractures are associated with medial collateral ligament rupture. In contrast, van Riet et al. [51] showed that associated ligamentous injury occurred in 11% of radial head fractures. In the present study, injuries to the lateral and medial collateral ligaments accounted for as many as 76.4% and 20.0% cases, respectively. These results are supported by the work of Kaas et al. [52] and Itamura et al. [53], who assessed injuries associated with radial head fractures using magnetic resonance imaging. The authors emphasized that magnetic resonance imaging allows for a better understanding of the mechanism of injury and the planning of appropriate treatment. In the present study, magnetic resonance imaging was not routinely performed when radial head fractures were diagnosed. Associated injuries were identified during surgery.

Harbrecht et al. [54] presented a case series of radial head fracture nonunion caused by interrupting cartilage fragments released from capitulum. In our study, capitulum fracture (21.8%) and exfoliation of radial head cartilage (16.4%) were also observed but did not significantly affect the results of treatment.

In terrible triad of the elbow, apart from a radial head fracture, there is a coronoid process fracture with dislocation of the elbow. The treatment of associated injuries is even more difficult and complex and can affect the final results of the treatment—both functionally and radiologically. This is the reason why we excluded this group of patients from the study. The same argument applies to the Monteggia fracture, but in most cases radial head is luxated, not broken. Moreover, at the time of the study, a small group of patients with these injuries (‘terrible triad’ n = 19, Monteggia fracture n = 5) were treated in the Department of Traumatology and Orthopedics, the results of statistical analysis would have been unrepresentative.

In the present article, no statistically significant differences were obtained in the clinical, functional or radiological results. This means that the treatment outcomes are not determined solely by the type of fracture. Instead, factors such as the quality of reduction, stable fixation, and associated injuries (articular cartilage damage, ligamentous rupture, accompanying capitulum fracture, etc.) play an important role. The experience of the surgeon and the use of more appropriate implants are also important [38]. Besides, ORIF is a technically more demanding procedure to perform compared to RHA [11]. Furthermore, according to Jakobi et al. [55], when ORIF methods fails, conversion to secondary prosthesis of the radial head yields good results.

The present study has several limitations. First, it was retrospective in nature and did not include a control group. To our knowledge, only one prospective study has evaluated the results of the treatment of radial head fractures using ORIF [32]. Second, the small sample size of the study group may have significantly affected the statistical analysis. It was also impossible to perform a comparative analysis; neither radial head resections nor arthroplasty was performed at the study center. Third, in some cases, it was impossible to obtain complete data from the in-hospital information systems. Fourth, the study did not consider concomitant fractures in the upper limbs except capitulum fracture (e.g., Monteggia fractures and terrible triad of the elbow). According to Pike et al., associated fractures can affect functional outcomes of radial head fractures treatment [35]. Fifth, the follow-up duration varied widely. The longer the time that has passed since the fracture, the more advanced adaptive processes that occur in the functionality of the limb. However, the long-term increasing risk of post-traumatic osteoarthritis must be considered.

## 5. Conclusions

Types II, III, and IV radial head fractures, according to the Mason classification, treated with open reduction and internal fixation provide good clinical results. Complications such as osteoarthritis, periarticular ossification and nonunion are rare. These fracture types should always be considered as complex injuries (articular cartilage and ligament rupture). The surgeon’s skills, preferences, and appropriate hardware are very important factors in determining good clinical and radiological outcomes of ORIF.

## Figures and Tables

**Table 1:** General characteristics of patients.

Variable	n	n (%)/M ± SD
Age, years	53	43.5 ± 15.1
Sex	53	
K		30 (56.6)
M		23 (43.4)
Nicotinism	53	5 (9.4)
BMI, kg/m^2^	32	28.4 ± 5.9
Occupation	46	
Office work		20 (45.5)
Services		8 (18.2)
Industry/crafts		2 (4.5)
Agriculture		2 (4.5)
Armed forces		1 (2.3)
Pensioner		6 (13.6)
Unemployed		5 (11.4)
Sports activity	44	24 (57.1)
Mechanism	55	
Fall on extended limb		34 (61.8)
Fall on a limb bent at the elbow joint		1 (1.8)
Low-energy traffic accident		11 (20.0)
High-energy traffic accident		4 (7.3)
Fall from a height		5 (9.1)
Dominant limb	51	24 (47.1)

M ± SD, mean ± standard deviation; M, males; K, females.

**Table 2:** Characteristics of the group during the surgical period with breakdown by fracture type.

Variable	Mason Type II (n = 15)	Mason Type III (n = 26)	Mason Type IV (n = 14)	*p*
Number of fragments				
2	11 (73.3)	5 (19.2)	5 (35.7)	0.002 ^2^
≥3	4 (26.7)	21 (80.8)	9 (64.3)	
Degeneration of articular cartilage	2 (13.3)	5 (19.2)	2 (14.3)	>0.999
LCL complex injury	11 (73.3)	18 (69.2)	13 (92.9)	>0.999
MCL injury	2 (13.3)	2 (7.7)	7 (50.0)	0.615
Capitulum fracture	3 (20.0)	6 (23.1)	3 (21.4)	>0.999
Neurovascular injuries	1 (6.7)	0 (0.0)	0 (0.0)	0.366
Immobilization time, days, median (IQR) *	14.5 (13.0; 30.3)	18.5 (14.0; 24.0)	23.0 (15.3; 27.0)	0.876 ^1^
Type of fixation				
Cannulated screw + plate	9 (60.0)	23 (88.5)	10 (71.4)	0.073
Cannulated screw only	5 (33.3)	2 (7.7)	4 (28.6)	
Plate only	1 (6.7)	1 (3.8)	0 (0.0)	

IQR; interquartile range; LCL lateral collateral ligament complex; MCL, medial collateral ligament. Comparisons were made using Kruskal-Wall’s test ^1^, Pearson’s chi-square test ^2^, and Fisher’s exact test. * Counted for group sizes: Type II, n = 14, Type III, n = 26, Type IV, n = 12.

**Table 3:** Comparison of treatment effects according to fracture type using the modified Mason scale.

Variable	Mason type II (n = 9)	Mason type III (n = 21)	Mason type IV (n = 11)	*p*
Interview				
Follow-up time [months], M ± SD	23.2 ± 7.8	20.8 ± 10.5	19.6 ± 11.9	0.730 ^1^
VAS, median (IQR)	4.0 (0.0; 8.0)	6.0 (0.0; 26.0)	5.0 (1.0; 13.0)	0.825 ^2^
Broberg-Morrey, M ± SD	82.0 ± 15.2	80.9 ± 15.5	84.2 ± 15.1	0.845 ^1^
DASH, median (IQR)	10.0 (5.0; 31.7)	11.7 (3.3; 25.0)	17.5 (6.3; 25.9)	0.937 ^2^
Physical examination				
Flexion, M ± SD	133.3 ± 12.3	137.1 ± 12.3	130.9 ± 11.4	0.366 ^1^
Extension deficit, median (IQR)	0.0 (0.0; 10.0)	5.0 (0.0; 20.0)	10.0 (5.0; 30.0)	0.076 ^2^
Flexion-extension arch, median (IQR)	130.0 (125.0; 140.0)	130.0 (110.0; 145.0)	120.0 (100.0; 127.5)	0.144 ^2^
Pronation, median (IQR)	90.0 (90.0; 90.0)	90.0 (70.0; 90.0)	80.0 (65.0; 90.0)	0.186 ^2^
Supination, median (IQR)	80.0 (70.0; 80.0)	60.0 (20.0; 70.0)	70.0 (60.0; 85.0)	0.051 ^2^
Arch pronation-supination, median (IQR)	160.0 (150.0; 170.0)	140.0 (100.0; 150.0)	140.0 (130.0; 160.0)	0.141 ^2^
Grip strength oper./contra., M ± SD	80.9 ± 23.4	81.7 ± 26.7	77.4 ± 22.9	0.896 ^1^
Lateral stability				
Total	9 (100.0)	21 (100.0)	11 (100.0)	>0.999
Medial stability				
Total	9 (100.0)	21 (100.0)	10 (90.9)	-
Partial	0 (0.0)	0 (0.0)	1 (9.1)	0.287
X-RAY *				
Bone union	9 (100.0)	19 (95.0)	11 (100.0)	>0.999
Displacement	0 (0.0)	0 (0.0)	0 (0.0)	-
Elbow osteoarthritis	1 (11.1)	3 (15.0)	2 (18.2)	>0.999
Ossifications according to the Hastings-Graham scale				
0	6 (66.7)	11 (55.0)	5 (45.5)	0.725
1	1 (11.1)	2 (10.0)	3 (27.3)	-
2A	1 (11.1)	0 (0.0)	2 (18.2)	-
2B	0 (0.0)	3 (15.0)	0 (0.0)	-
2C	1 (11.1)	3 (15.0)	1 (9.1)	-
3A	0 (0.0)	1 (5.0)	0 (0.0)	-
Avascular necrosis of the radial head	0 (0.0)	1 (5.0)	0 (0.0)	>0.999

M, mean; SD, standard deviation; IQR, interquartile range. Comparisons were made using Anova analysis ^1^, Kruskal-Wallis test ^2^ and Fisher’s exact test. * X-ray parameters available for group sizes: Mason type II, n = 9, Mason type III, n = 20, Mason type IV, n = 11.

**Table 4:** Clinical outcomes by fracture type.

Outcome	Mason Type II (n = 9)	Mason Type III (n = 21)	Mason Type IV (n = 11)
Very good	4 (44.4)	8 (38.1)	5 (45.5)
Good	4 (44.4)	9 (42.9)	4 (36.4)
Fair	0 (0.0)	3 (14.3)	2 (18.2)
Bad	1 (11.1)	1 (4.8)	0 (0.0)

## Data Availability

Data are contained within the article.

## Abbreviations

The following abbreviations are used in this manuscript:

AMMS	Asseco Medical Management Solutions
AP	anteroposterior view
BMI	Body Mass Index
CT	Computed Tomography
DASH	Disabilities of Arm, Shoulder, and Hand Questionnaire
IQR	interquartile range
K	females
LCL	lateral collateral ligament
M	males
M ± SD	mean ± standard deviation
MCL	medial collateral ligament
ORIF	Open Reduction and Internal Fixation
RHA	Radial Head Arthroplasty
ROM	range of motion
VAS	Visual Analog Scale
